# The clinical regimens and cell membrane camouflaged nanodrug delivery systems in hematologic malignancies treatment

**DOI:** 10.3389/fphar.2024.1376955

**Published:** 2024-04-16

**Authors:** Yuanyuan Liu, Shanwu Yu, Yixiang Chen, Zhihong Hu, Lingling Fan, Gaofeng Liang

**Affiliations:** ^1^ College of Basic Medicine and Forensic Medicine, Henan University of Science and Technology, Luoyang, Henan, China; ^2^ College of Horticulture and Plant Protection, Henan University of Science and Technology, Luoyang, Henan, China; ^3^ Luoyang Vocational and Technical College, Luoyang, Henan, China

**Keywords:** hematologic malignancies, clinical regimens, nanocarrier, nanodrug delivery system, cell membrane camouflage

## Abstract

Hematologic malignancies (HMs), also referred to as hematological or blood cancers, pose significant threats to patients as they impact the blood, bone marrow, and lymphatic system. Despite significant clinical strategies using chemotherapy, radiotherapy, stem cell transplantation, targeted molecular therapy, or immunotherapy, the five-year overall survival of patients with HMs is still low. Fortunately, recent studies demonstrate that the nanodrug delivery system holds the potential to address these challenges and foster effective anti-HMs with precise treatment. In particular, cell membrane camouflaged nanodrug offers enhanced drug targeting, reduced toxicity and side effects, and/or improved immune response to HMs. This review firstly introduces the merits and demerits of clinical strategies in HMs treatment, and then summarizes the types, advantages, and disadvantages of current nanocarriers helping drug delivery in HMs treatment. Furthermore, the types, functions, and mechanisms of cell membrane fragments that help nanodrugs specifically targeted to and accumulate in HM lesions are introduced in detail. Finally, suggestions are given about their clinical translation and future designs on the surface of nanodrugs with multiple functions to improve therapeutic efficiency for cancers.

## 1 Introduction

**FIGURE 1:**
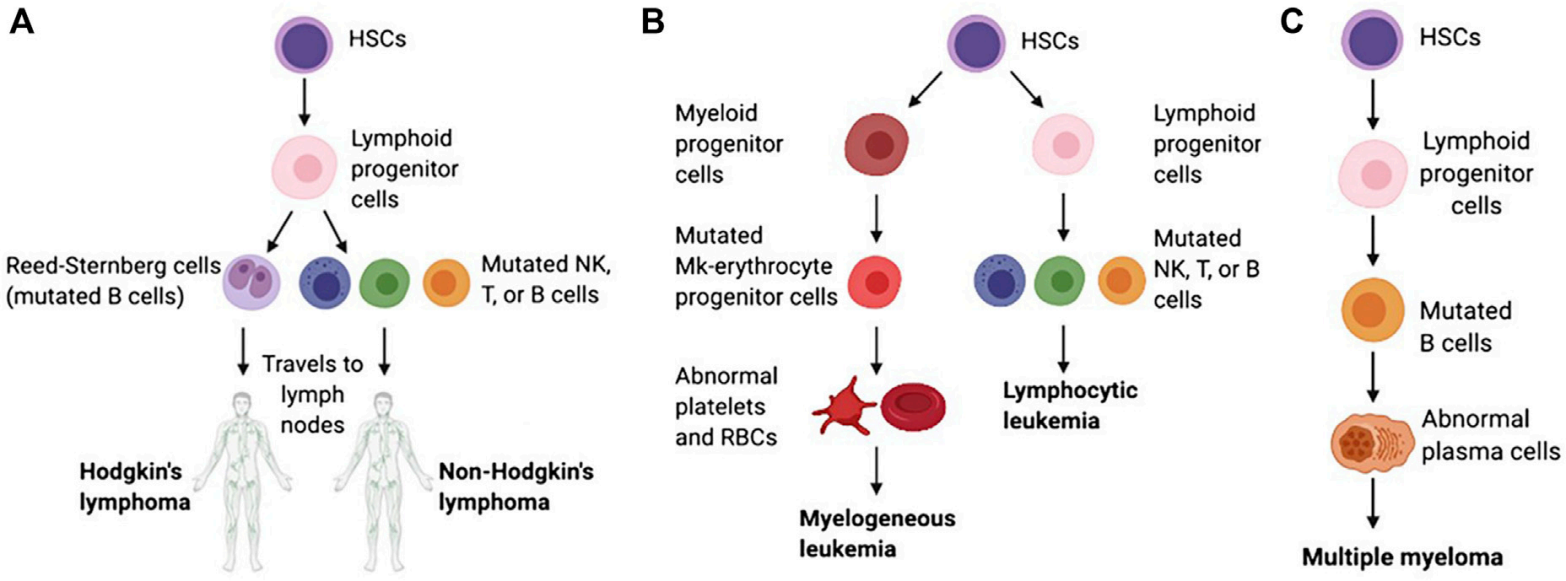
Schematic of the pathological differentiation of lymphoma **(A)**, leukemia **(B)** and MM **(C)**, copy from (Powsner et al., 2021) with license under CC BY 4.0.

Hematologic malignancies (HMs), which are also called blood cancers, often begin in the cells of the immune system or in blood-forming tissue such as the bone marrow, where stem cells develop into blood cells, affecting the bone marrow’s ability to make enough blood cells (red blood cells, white blood cells and platelets) (Greim et al., 2014). When abnormal HM cells grow uncontrollably, they can cause cancers to develop, outpacing the growth of healthy blood cells and disrupting their normal functions. According to the first detected sites, the HMs are traditionally categorized as leukemias (blood), lymphomas (lymph nodes), or myelomas (bone) (Bispo et al., 2020; Alaggio et al., 2022) (Figure 1).

### 1.1 Leukemia

Leukemia begins in early blood-forming cells in the bone marrow, the spongy tissue inside bones (Mehranfar et al., 2017). The type of leukemia depends on the type of blood cell that has become cancerous. For instance, acute lymphoblastic leukemia (ALL) is a cancer of the lymphoblasts, white blood cells that fight infection. White blood cells are the most common type of blood cell to become cancerous (lymphocytic leukemia). However, red blood cells, which carry oxygen from the lungs to the rest of the body, and platelets, which clot the blood, may also become cancerous (myelogeneous leukemia). Common leukemias include ALL, chronic lymphoblastic leukemia (CLL), acute myeloid leukemia (AML), chronic myeloid leukemia (CML), chronic myelomonocytic leukemia (CMML), chronic neutrophilic leukemia (CNL) and atypical chronic myeloid leukemia (aCML), etc (Li et al., 2014; Dao et al., 2017).

### 1.2 Lymphoma

Lymphoma begins in lymphocytes and white blood cells that are part of the lymphatic system, which is part of the immune system and helps fight infection and disease (McLafferty et al., 2012). Lymphoma can begin almost anywhere in the body because lymph tissue exists everywhere. The lymphomas mainly include non-Hodgkin’s lymphoma (NHL, containing T, NK, and B cell tumors) and Hodgkin’s lymphoma (HL) (Di Pietro and Good-Jacobson, 2018). Lymphomas occur in both children and adults.

### 1.3 Myeloma

Myeloma begins with the abnormal development of plasma cells that make antibodies that help the immune system fight infections and disease in bone marrow or soft tissue (Lee and Borrello, 2016). When there is only one tumor, the disease is referred to as plasmacytoma. When there are multiple tumors, it is known as multiple myeloma (MM). Both conditions are malignant (cancerous). Common myelomas include MM, myeloproliferative disorder (MPD), myelodysplastic syndrome (MDS), myelodysplastic/myeloproliferative disorder (MD/MPD), etc (Pati and Kundil Veetil, 2019).

The clinical strategies for treating HMs include radiotherapy (Dabaja and Spiotto, 2023), chemotherapy (Atkins and He, 2019), targeted molecular therapy (Shimada, 2019), hematopoietic stem cell transplantation (HSCT) (Sivakumar et al., 2019), immunotherapy (Kansara and Speziali, 2020), bloodless transplantation for populations who cannot receive blood products (Scharman et al., 2017) and their combinations (Wu and Singh, 2011). Radiotherapy and chemotherapy are the most traditional HMs therapeutic strategies with significant limitations owing to strong side effects (Liu et al., 2021). Targeted molecular therapy supplies higher safety and precision for HMs treatment, and some inhibitors have been approved for marketing. However, it is a pity that the low stability and off-targeting toxicity of targeted drugs limited their therapeutic effect for HMs (Huang L. et al., 2020). The HSCT program injects healthy stem cells into the blood to replace diseased bone marrow, and the new bone marrow makes the healthy cells that the body needs and helps slow or stop blood cancers (Burt et al., 2008). However, the HSCT strategy is usually unenforceable because of a need for adequately matched bone marrow (Fenwarth et al., 2021). Immunotherapy, a hot spot of clinical HMs treatment in recent years, also possess some drawbacks such as resistance to checkpoint inhibitors, causing cytokine release syndrome, etc (van de Donk and Usmani, 2018; Wei et al., 2020). Even though the 5-year survival of HMs patients has improved with various advanced clinical strategies, the average survival and cure rates are still low (Hemminki et al., 2023). Therefore, more effective methods are worth exploring to optimize HMs treatment.

The nanodrug delivery system is an excellent method to help resolve drug delivery problems in HMs treatment. The inherent physical and chemical properties of drugs including poor solubility, low stability, and no targeting ability, lead to side effects on normal tissues in the body, often limiting their therapeutic effect in clinical application (Halwani, 2022). Various nanocarriers have been developed to carry anti-HMs drugs reaching HMs lesions with focused accumulation, controlled drug release, and low side effects (Jia et al., 2023). Among nanocarriers, liposome is the U.S. Food and Drug Administration (FDA)-approved nanocarrier for drug delivery against HMs (Hani et al., 2023). The targeting accumulation of nanodrugs is a crucial factor guaranteeing drug work in HMs lesions rather than other normal tissues, and this aim is usually achieved by a decoration of targeted ligands or cell membrane camouflage on the surface of nanodrug (Deshantri et al., 2018). In this review, we will first introduce the common types of nanocarriers, and then submit in detail the application of targeting strategies for nanodrug delivery systems in HMs treatment. Finally, we will discuss the prospects and challenges of targeting decoration for nanodrug delivery systems.

## 2 The clinical regimens for the treatment of HMs

### 2.1 Radiotherapy

Radiotherapy employs high-energy beams or particles, such as X-rays, gamma rays, and charged particles, to directly damage cancer cells’ DNA or generate charged particles (free radicals) within the cells, ultimately disrupting their DNA and causing cell death (Baskar et al., 2014). Radiotherapy is generally performed with high doses targeted to a localized region to damage DNA. The advantages of radiotherapy include the complete elimination of tumors in areas where conventional surgery is not feasible, along with minimal adverse effects. The approach to radiotherapy for HMs hinges on the specific locations of cancer manifestation within the body. Radiotherapy treats HMs by eliminating cancer cells in the bloodstream, providing relief from pain or discomfort resulting from an enlarged liver, spleen, or swollen lymph nodes. Additionally, it is employed to alleviate pain stemming from bone damage caused by the proliferation of cancer cells in the bone marrow. Some specialized radiotherapy techniques for HMs include total body irradiation for leukemia patients, total skin electron beam therapy for treating mycosis fungoides (a type of T-cell lymphoma), cranio-spinal radiation for leukemia patients with central nervous system involvement, and involved site radiation therapy for treating HL, etc. However, dose-related toxicity that occurs frequently due to damage to the surrounding healthy tissue near the target site often poses a limitation. Given the widespread distribution of malignant cells in HM, radiotherapy only proves effective in a small proportion of patients.

Driven by the need to prevent long-term toxicity that elevates morbidity and mortality among long-term survivors, radiation fields and doses have been reduced (Radiation for hematologic malignancies: from cell killing to immune cell priming). Nowadays, low-dose radiation like 4 Gy is also successfully used as a curative modality for various HMs in clinical treatment (Ciammella et al., 2018; Imber et al., 2021). Low-dose radiation can activate cellular defense mechanisms that repair DNA damage, remove cells via autophagy and apoptosis that cannot be repaired, and trigger cell cycle arrest to prevent damaged cells from dividing and allowing for repair (Tharmalingam et al., 2019). In addition, it can also induce adaptive memory, protecting against future oxidative stress and enhancing the immune system (Farooque et al., 2011; Dabaja and Spiotto, 2023). The anti-tumor immune responses through low-dose radiation are ushering in a transformative era for radiotherapy in the treatment of HMs, bolstering the efficacy of immunotherapy and adoptive cell-based therapy.

### 2.2 Chemotherapy

Chemotherapy is a based therapeutic strategy for HMs. Because of lack of non-specifically targets, chemotherapy can affect the entire body of HMs patients, and hence can destroy metastasized cancer cells (Nygren, 2001; Chu and Sartorelli, 2018). FDA-approved chemo-drugs in HMs treatment include DNA-interactive agents such as chlormethine, bleomycin, doxorubicin, cisplatin, cyclophosphamide, daunorubicin, cytarabine, and melphalan; antimetabolites such as gemcitabine, methotrexate, fluorouracil and mercaptopurine; anti-tubulin agents like vincristine, etc (Powsner et al., 2021; Sochacka-Ćwikła et al., 2021). The time-honored first-line therapy known as the “3 + 7” regimen (daunorubicin and cytarabine) for leukemia has been a mainstay in clinical practice for over five decades, since 1973 (Li J. et al., 2023). In addition, combined utility of chemo-drugs with different mechanisms can kill more cancer cells by reducing the likelihood of cancer becoming resistant to any chemo-drug (Housman et al., 2014). However, because chemo-drugs can kill cancerous and healthy cells, which leads to patients usually experiencing side effects from treatment, hence it cannot bring about the ideal therapeutic effect (Van den Berg et al., 2017; Oun et al., 2018).

### 2.3 Hematopoietic stem cell transplantation (HSCT)

**FIGURE 2:**
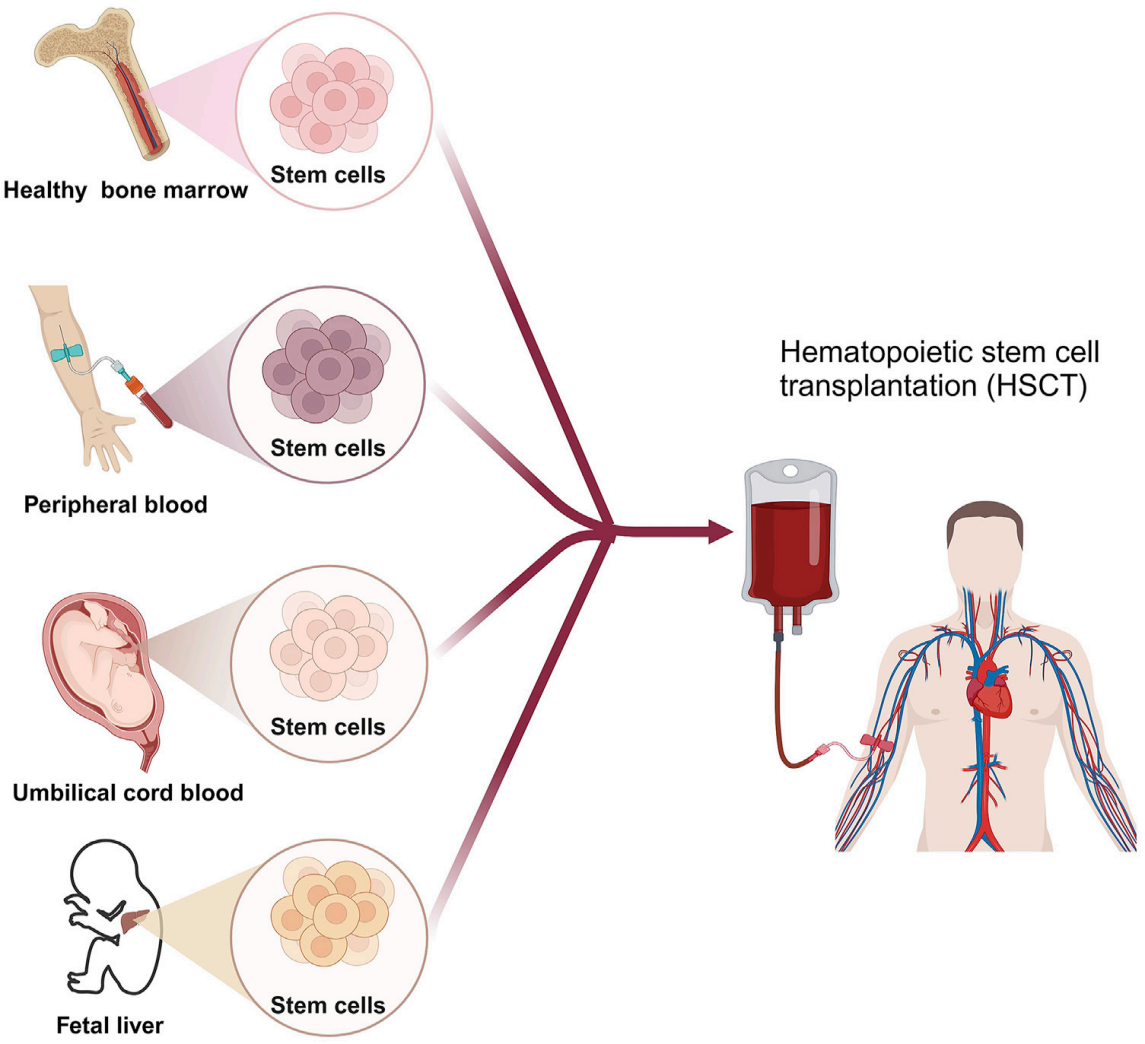
The scheme of HSCT for HMs patient. Created by BioRender (agreement number: NT26D16T7T).

HSCT involves the intravenous infusion of hematopoietic stem cells to restore blood cell production in patients with damaged or defective bone marrow or immune systems (Chivu-Economescu and Rubach, 2017). HSCT cells can be obtained from the patient themselves (autologous transplant), another person such as a sibling or unrelated donor (allogeneic transplant), or an identical twin (syngeneic transplant) (Giralt and Bishop, 2009; Kurosawa et al., 2021). Cell sources consist of bone marrow, peripheral blood, umbilical cord blood, or rarely, fetal liver (Saulnier et al., 2005) (Figure 2). The HSCT process is mainly divided into 5 phases sequentially, including conditioning, stem cell infusion, neutropenic phase, engraftment phase, and post-engraftment period (Hatzimichael and Tuthill, 2010). More than half of autologous transplantations are conducted for the treatment of MM and NHL (Costa et al., 2015; Bhatt, 2016), while the majority of allogeneic transplants are performed for hematologic and lymphoid cancers (Majhail et al., 2015). Autologous transplantation provides rapid recovery of patient cell counts, reduces transplant-related morbidity, shortens hospitalization periods, and is cost-effective compared to allogeneic grafts. A significant advantage of an allogeneic graft is the powerful contribution of the donor’s immune system in eradicating cancer through the graft-versus-tumor effect. However, an essential barrier to HSCT in clinical application is the inability to secure suitable donors (Riezzo et al., 2017). In addition, relapse is frequently unavoidable in autologous transplantations due to the contamination of transplanted cells with cancer cells. While allogeneic HSCT circumvents relapse triggered by autologous HSCT, the diverse immune responses (graft vs. host disease) stemming from heterogeneity pose a significant obstacle to its effectiveness in treating HMs.

### 2.4 Targeted molecular therapy

The significant molecular heterogeneity present in HMs poses major challenges for precision medicine and the development of tailored treatment approaches. Many target sites have been developed for the treatment of HMs due to genetic or epigenetic carcinogenic mutations. Monoclonal antibodies and small selective molecules are usually used to inhibit carcinogenic mutations in HMs. Unlike chemotherapy, targeted molecular therapy has high selectivity and precision for cancer cells, thus resulting in lower toxic and side effects to healthy tissues in the body. Common types of targets mediating targeted molecular therapy in HMs are exhibited as below.

#### 2.4.1 Protein tyrosine kinase (PTK)

PTK Mutations can result in aberrant or overexpressed PTK activity, leading to dysregulated cell signaling pathways, unchecked cell proliferation, and evasion of cell death mechanisms in various types of HMs. PTK is a family of kinases that catalyze the transfer of γ-phosphate from ATP to protein tyrosine residues. A common feature is the presence of a typical PTK domain at the carboxyl terminus, which catalyzes the phosphorylation of itself or substrates and plays an vital role in cell growth, proliferation, and differentiation (Schenk and Snaar-Jagalska, 1999; Paul and Mukhopadhyay, 2004). Most of the tyrosine kinases discovered to date are oncogene products belonging to oncogenic RNA viruses, and can also be produced by proto-oncogenes in vertebrates (Parisi et al., 2023). The PTKs are mainly divided into receptor tyrosine kinases (RTKs) and non-receptor tyrosine kinases (nRTKs) (Jiao et al., 2018). Mutations in the FMS-like tyrosine kinase-3 (FLT3) gene, particularly internal tandem duplications (ITD) and point mutations in the tyrosine kinase domain, are frequently observed in AML. These mutations dysregulate FLT3 signaling, thereby fostering cellular survival and proliferation (Kiyoi et al., 2020). Tyrosine-protein kinase KIT (c-KIT) mutations are observed in various HMs, and almost 80% of AML has KIT proto-oncogene expression (Ikeda et al., 1991). BCR-ABL tyrosine kinase mutation, typically resulting from a reciprocal translocation between chromosomes 9 and 22 to produce the BCR-ABL oncogene, is found in 90% of CML and a subset of ALL, and are more common in adults than children. This protein has constitutive tyrosine kinase activity that activates numerous downstream pathways that ultimately produces uncontrolled myeloid proliferation and leads to constitutive activation of tyrosine kinase activity, driving uncontrolled proliferation of myeloid cells (Arana-Trejo et al., 2002; Leoni and Biondi, 2015). The mutation of Bruton’s tyrosine kinase (BTK) serves as a significant contributor to B-cell lymphomas and leukemias, playing a pivotal role in their pathogenesis and progression by facilitating abnormal proliferation and survival of B-cells (Pal Singh et al., 2018). Mutations in the spleen tyrosine kinase (SYK) gene, a crucial component of B-cell receptor (BCR) signaling, have been implicated in the pathogenesis of lymphomas and leukemias. These mutations can dysregulate SYK activity, leading to aberrant proliferation, survival, and differentiation of B-cells, which play a pivotal role in the development and activation of these cells. Consequently, these alterations contribute to the development of hematological cancers (Kutsch et al., 2015). Common tyrosine kinase inhibitors (TKIs) marketed or in the progress of clinical trials in HMs treatment are shown in Table 1.

**TABLE 1:** Common TKIs in HMs treatment (from www.clinicaltrials.gov).

NCT number	PTK	TKI	Status	Phase	Conditions
NCT05563545	FLT3	Gelteritinib	Marketing		AML
NCT02474290	FLT3	Sorafenib	Completed	Phase 2/3	AML
NCT01830361	FLT3	Midostaurin	Completed	Phase 2	AML
NCT03735875	FLT3	Quizartinib	Completed	Phase 1/2	AML
NCT03070093	FLT3	Gilteritinib	Marketing		AML
NCT02400255	FLT3	Crenolanib	Completed	Phase 2	AML
NCT01349049	FLT3	Pexidartinib	Completed	Phase 1/2	AML
NCT03580655	c-KIT	Avapritinib	Active, not recruiting	Phase 2	Mast Cell Leukemia
NCT01126814	c-KIT	Imatinib	Completed	Phase 1/2	Leukemia
NCT00130195	BCR-ABL	Imatinib	Completed	Phase 2	ALL
NCT04155411	BCR-ABL	Dasatinib	Recruiting	Phase 4	CML
NCT01535391	BCR-ABL	Nilotinib	Completed	Phase 3	CML
NCT04070443	BCR-ABL	Ponatinib	Active, not recruiting	Phase 2	CML
NCT03128411	BCR-ABL	Bosutinib	Completed	Phase 2	CML
NCT04662255	BTK	Ibrutinib	Recruiting	Phase 3	MCL
NCT02029443	BTK	Acalabrutinib	Active, not recruiting	Phase 1/2	CLL, Small Lymphocytic Lymphoma (SLL)
NCT04116437	BTK	Zanubrutinib	Recruiting	Phase 2	CLL, MCL, SLL
NCT03162536	BTK	Tirabrutinib	Active, not recruiting	Phase 1/2	SLL, CLL, MCL
NCT05030675	SYK	Fostamatinib	Recruiting	Phase 1	CML, MDS
NCT03010358	SYK	Entospletinib	Completed	Phase 1/2	MCL, SLL, CLL

#### 2.4.2 Isocitrate dehydrogenase (IDH)

IDH mutation are frequently found in certain types of leukemia, such as AML and MDS. The IDH is an essential metabolic enzyme in the Krebs Cycle and its families include IDH1 and IDH2 (Upadhyay et al., 2017). The conserved mutational hotspot of IDH1 is R132, and of IDH2 are R140 and R172. IDH mutant forms transformed α-KG into 2-hydroxyglutarate (2-HG), which induced a number of malignancies like inhibits DNA demethylases and histone demethylases (Janin et al., 2014; Mondesir et al., 2016). IDH mutation are frequently found in certain types of leukemia, such as AML and myelodysplastic syndromes (MDS). Ivosidenib (mutant IDH1 inhibitor) (Norsworthy et al., 2019) and enasidenib (mutant IDH2 inhibitor) (Fathi et al., 2020) are currently available IDH inhibitors for clinically treating AML, and ivosidenib became the first targeted drug approved to treat relapsed/refractory (R/R) MDS with IDH1 mutations in 2023.

#### 2.4.3 B-cell lymphoma-2 (BCL-2) family

The BCL-2 family of proto-oncogenes plays a pivotal role in regulating apoptosis, the programmed cell death process, and their dysregulated expression is a hallmark of various human cancers, notably prevalent in certain lymphomas and leukemias. The BCL-2 family, including BCL-2, BCL-B, BCL-X_L_, BCL-W, myeloid cell leukemia sequence 1 (MCL-1) and BFL1, is a crucial regulator of apoptosis triggered by various environmental and stress signals (Cory and Adams, 2002). Anti-apoptotic protein BCL-2 is usually overexpressed in leukemias and lymphomas, leading to the occurrence and development of tumors, and limiting their response to chemotherapy (Roberts, 2020). BH3-mimetics including ABT-737 (Kuroda et al., 2006), ABT-263 (Kipps et al., 2015), and Venetoclax (Roberts et al., 2016), a class of targeted drugs that mimic the actions of BH3-only proteins binding to BCL-2 and hence inhibit BCL-2’s ability, are popular for clinical research in treating leukemias and lymphomas. MCL-1 is another important antiapoptotic protein in promoting cell survival and drug resistance in MM, AML, and NHL. Therefore, some MCL-1 inhibitors including AZD5991, S64315, AMG 176, and AMG 397, are also in clinical trials in HMs treatment.

#### 2.4.4 Phosphatidylinositol-3-kinases (PI3Ks)

PI3Ks inhibition often mark significant progress in CLL and related malignancies. PI3Ks, a family of lipid kinases, are categorized into three main classes (I, II, and III) based on their structure and substrate specificity. Among these, Class I PI3Ks, which include p110α, p110β, p110δ, and p110γ, are most closely linked to human cancer. In 2014, a milestone was reached when idelalisib, targeting p110δ, became the first FDA-approved PI3K inhibitor. It gained approval for treating relapsed follicular lymphoma (FL), SLL, and CLL (Wei et al., 2015). Another notable PI3K inhibitor is duvelisib, an oral medication that inhibits both p110γ and p110δ. It received FDA approval in 2018 specifically for CLL patients who have undergone at least two prior therapies (Flinn et al., 2018). Ongoing clinical investigations are exploring other PI3K inhibitors targeting p110δ for CLL therapy. Prominent examples include BGB-10188, HMPL-689, parsaclisib, umbralisib, and zandelisib. Moreover, copanlisib and buparlisib are PI3K inhibitors with a broader spectrum, simultaneously targeting p110α, p110β, p110δ, and p110γ (Hus et al., 2022).

#### 2.4.5 Epigenetic mutations

Epigenetic modifier gene mutations are common in HMs, playing a pivotal role in both the onset and advancement of cancer. As reported, some epigenetic changes such as DNA methylation-related mutations, abnormal histone deacetylase (HDAC) and bromodomain and extraterminal (BET) protein expression are recurrent in HMs (Zhao et al., 2023). Drugs targeting epigenetic changes have made clinical progress in treating HMs. Clinical DNA hypomethylating agents (HMAs) in HMs therapy mainly include azacitidine (Odenike, 2017), decitabine (Santos et al., 2010), guadecitabine (Kantarjian et al., 2017) and ASTX727 (cedazuridine/decitabine) (Abuasab et al., 2022). Common HDAC inhibitors used for clinical trials in HMs treatment include vorinostat (Hopfinger et al., 2014), belinostat (Johnston et al., 2021), panobinostat (El Omari et al., 2023), chidamide (Cao et al., 2023), and romidepsin (Bates et al., 2015). As the core member of the BET family, BRD4 regulates gene expression, and its inhibitors, such as JQ1 (Pericole et al., 2019) and AZD5153 (Rhyasen et al., 2016), are also potential clinical drugs in HMs therapy.

### 2.5 Immunotherapy

The continuous interaction between immune cells and cancer cells within the hematopoietic system creates an environment conducive to immune surveillance. Since malignancies originate from the same cellular sources as the immune system, cancer cells possess immunostimulatory characteristics. However, this dual nature can also lead to compromised immune responses. Significant progress has been made in cancer immunotherapy, with accelerating advancements based on diverse strategies aimed at harnessing the host immune system. In the realm of hematologic malignancies treatment, immunotherapy primarily encompasses targeted antibodies, immune checkpoint blockade (ICB), and CAR cell therapy.

#### 2.5.1 Monoclonal antibodies

Monoclonal antibodies (mAbs) stand as a cornerstone in cancer immunotherapy. mAbs are precisely uniform IgG antibodies derived from a single B cell clone, specifically targeting unique antigenic epitopes (Weiner et al., 2009). With diverse mechanisms of action, each type of antibody engages multiple facets of immunity, orchestrating a comprehensive assault on tumor cells. mAbs can be directed against tumor-associated antigens (TAAs) and killing cancer cells through two primary pathways: i) direct induction of apoptosis via programmed cell death (PCD) (Overdijk et al., 2016); ii) immune-mediated mechanisms, notably including antibody-dependent cellular cytotoxicity (ADCC), complement-dependent cytotoxicity (CDC), and antibody-dependent macrophage-mediated phagocytosis, facilitated by the interaction between Fc and FcγR (Fc gamma receptor) (Tipton et al., 2015; Krejcik et al., 2016).

Rituximab, ofatumumab, obinutuzumab, and ibritumomab tiuxetan are FDA-approved mAbs that bind to CD20 antigens expressed on the surface of immune system B cells, subsequently eradicating the HMs such as CLL, diffuse large B-cell lymphoma (DLBCL), B cell NHL (B-NHL), and follicular lymphoma (FL), etc (Wierda et al., 2010; Morschhauser et al., 2013; Goede et al., 2014; Salles et al., 2017; Pierpont et al., 2018). Daratumumab and isatuximab are FDA-approved anti-CD38 mAbs for MM immunotherapy because CD38, a type II transmembrane glycoprotein, is highly expressed in MM cells (Palumbo et al., 2016; Goldschmidt et al., 2022). Michel de Weers et al. found daratumumab not only exhibited potent cytotoxicity to CD38-expressing lymphoma- and MM-derived cell lines as well as in patient MM cells by Ab-dependent cellular cytotoxicity *in vitro*, but also highly activated and interrupted xenograft tumor growth at low dosing *in vivo* (De Weers et al., 2011). In addition, other types of mAbs have been approved by FDA for treating HMs including tafasitamab (anti-CD19 mAb) for DLBCL and FL (Salles et al., 2020), alemtuzumab (anti-CD52 mAb) for peripheral T-cell lymphoma (PTCL) and CLL (Wulf et al., 2021), elotuzumab (anti-CS1 mAb) for MM (Dimopoulos et al., 2023), and milatuzumab (anti-CD74 mAb) for MM, (MCL), FL and CLL (Kaufman et al., 2008; Alinari et al., 2009; Hertlein et al., 2010; Christian et al., 2015).

#### 2.5.2 Immune cell engagers

Some HMs treated with mAbs are often R/R. Fortunately, immune cell engagers (engineered antibodies) are designed and developed in treating R/R HMs with high efficiency in recent years (Tian et al., 2021). Immune cell engagers work by at least one arm recognizing TAAs and at least another one arm recruiting immune effector cells (CD3 for T cells and CD16a for NK cells). Therefore, immune cell engagers enable the direct targeting of immune cells to tumors, substantially mitigating resistance and serious adverse effects. Immune cell engagers are divided into bispecific antibodies (BsAbs), trispecific antibodies (TsAbs) and even tetraspecifc antibodies. Compared to BsAbs, TsAbs expand therapeutic options by introducing a third binding component. This additional moiety allows for the targeting of an extra tumor-associated antigen, enhancing specificity and preventing immune evasion. Alternatively, it can target additional costimulatory receptors on immune cells, thereby enhancing their effector functions (Liu D. et al., 2022). Until now, around 100 bispecific T cell engagers are in clinical trials, and NK cell engagers and TsAbs are currently undergoing early-stage clinical studies because of late starting (Tapia-Galisteo et al., 2023).

Blinatumomab is the first FDA-approved bispecific T cell engager, working by transiently linking CD19-positive B cells to CD3-positive T cells, resulting in induction of T-cell-mediated serial lysis of B cells and concomitant T-cell proliferation. It successfully treated Philadelphia chromosome-negative R/R B-cell precursor ALL (Zhu et al., 2016). Recent marketed T cell engagers for treating HMs include the anti-CD20×anti-CD3 mosunetuzumab for FL and epcoritamab for DLBCL, the anti-B cell maturation antigen (BCMA)×anti-CD3 teclistamab for MM, and gloftamab for DLBCL. In 2023, FDA approved BsAb talquetamab-tgvs (anti-G Protein-coupled Receptor class C group 5 member D×anti-CD3) and elranatamab-bcmm (anti-BCMA×anti-CD3) for adults with R/R MM who have received at least four prior lines of therapy, including a proteasome inhibitor, an immunomodulatory agent, and an anti-CD38 monoclonal antibody. Some NK cell engagers are in clinical trials including anti-CD30×anti-CD16a AFM13 for HL and NHL and anti-CD123×anti-CD16a AFM28 for ALM, etc. Some TsAbs and tetraspecifc antibodies are also in clinical stages, such as anti-CD38×anti-CD3×anti-CD28 SAR442257 for MM and NHL, anti-CD3×anti-CD137×anti-PD-L1×anti-CD19 emfzatamab for NHL, and anti-BCMA×anti-CD16×anti-Natural Killer Group 2 member D CC-92328 × DF3001 for R/R MM, etc (www.clinicaltrials.gov).

#### 2.5.3 Immune checkpoint blockade

Immune cells have two main mechanisms for killing tumor cells. The first is through specific signaling via immune cell receptors, and the second is through nonspecific signals. These nonspecific signals are linked to costimulatory receptors like CD28, or coinhibitory receptors like cytotoxic T lymphocyte-associated protein 4 (CTLA-4) and programmed cell death-1 (PD-1) (Chen and Flies, 2013). When coinhibitory receptors are engaged, cytotoxic T cells and NK cells are suppressed, allowing tumor cells to evade the immune system. Therefore, the utility of immune checkpoint inhibitors (ICIs) to block inhibitory checkpoints can harness immune cells to effectively attack tumor cells effectively.

Many anti-CTLA-4 (ipilumumab), anti-PD-1 (pembrolizumab, nivolumab, cemiplimab), and anti-PD-L1 (atezolizumab, avelumab, durvalumab) ICB drugs are in clinical trials for the treatment of HMs (Salik et al., 2020). Nivolumab and pembrolizumab have been approved by FDA for treating HL and primary mediastinal large B-cell lymphoma, respectively (Kroll et al., 2022). As a PD-1 inhibitor, pembrolizumab has recently been used in a phase IB clinical trial with R/R MM (Ribrag et al., 2019). CTLA4-blocking antibody ipilimumab provided substantial anti-tumor activity in patients with both lymphoid and myeloid malignancies after allogeneic HSCT (Davids et al., 2015). Matthew S. Davids et al. reported a prospective clinical phase 1 trial of PD-1 blockade using nivolumab for relapsed HMs after alloHCT (Davids et al., 2020). D Liao et al. utilized anti-PD1 or anti-CTLA4 inhibitor to treat AML patients under the stage of disease remission after chemotherapy, and results demonstrated strong T cell responses against residual AML (Liao et al., 2019). Other immune checkpoint molecules on T cells, such as LAG-3, TIM-3, and TIGIT also play an important role in inactivating T cell, and inhibition of them can be a potential approach to induce T cell responses in a non-redundant manner (Anderson, 2014; Blake et al., 2016; Maruhashi et al., 2018). In addition, NK cell ICIs such as anti-NKG2D (natural-killer group 2, member D) mAb and anti-KIR mAb also showed good clinical trials in HMs patients (Vey et al., 2012; André et al., 2018). Current hinders for ICB application in HMs treatment include cytokine-release syndrome, primary resistance, and acquired resistance, etc. Finding out new immune checkpoints and inhibiting resistance may improve the above problems.

#### 2.5.4 CAR cell therapy

**FIGURE 3:**
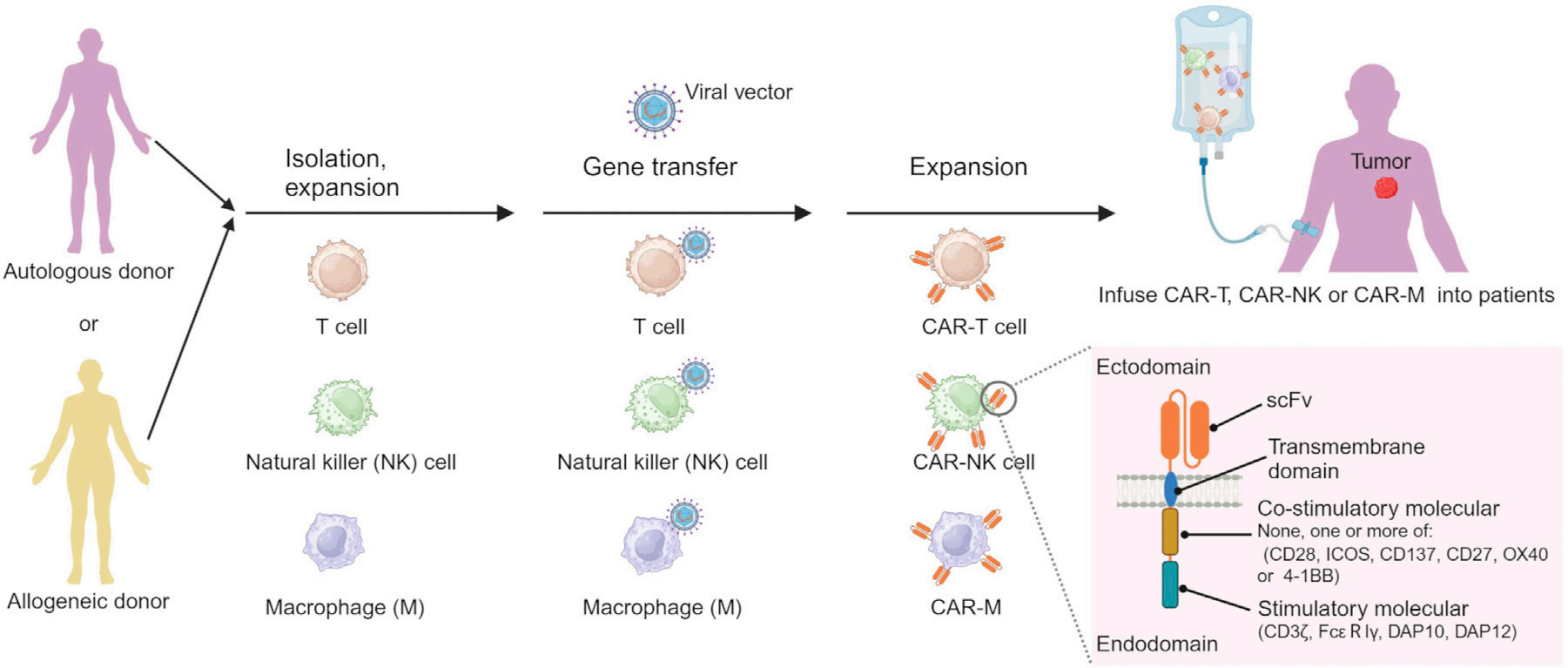
The processes of preparing CAR-immune cell. Created by BioRender (agreement number: TS26D15BJS).

CAR cell therapy stands out as one of the most promising immunotherapeutic approaches, exhibiting remarkable efficacy in the treatment of HMs and achieving significant advancements recently. Chimeric antigen receptor (CAR) cell therapies, including CAR-T cell, CAR-natural killer (CAR-NK) cell and CAR-macrophage (CAR-M) therapy, are novel forms of tumor immunotherapy (Boyiadzis et al., 2018). The CAR contains a single-chain variable fragment (scFv) that recognizes the tumor antigen at extracellular sites, a transmembrane domain derived from CD4, CD8α, CD28 or CD3ζ, none, one or more of intracellular co-stimulatory molecular such as CD28, ICOS, CD137, CD27, OX40 or/and 4-1BB, and an intracellular immune cell activation domain, such as CD3ζ chain, DAP10, DAP12 or FcεRIγ chain (Figure 3) (Khalil et al., 2016; Oberschmidt et al., 2017; Hu et al., 2018; Pfefferle and Huntington, 2020). The technology of CAR-T cell involves extracting T cells from a patient’s immune system, culturing and modifying them *in vitro*, equipping them with particular molecules to enable them to recognize and attack specific cancer cells, and then reintroducing the modified T cells into the patient’s body. The modified T cells become CAR-T cells, which travel throughout the body. Once these detectors receive specific signals from the surfaces of other cells, they activate the CAR-T cells and initiate an attack, eliminating the signal-carrying cells as adversaries (Han et al., 2021). Similar processes occur in CAR-NK and CAR-M (Figure 3) (Hu et al., 2018; Tariq et al., 2018; Klichinsky et al., 2020).

CD19, CD20, CD22, CD33, CD56, CD70, CD79b, CD5, CD7, CD123, BCMA, and NKG2DL are the common targets of CAR cell therapy, and their application in clinical trials in HM patients, as shown in Table 2. So far, FDA-approved CAR cell medications are CAR-T cells targeting CD19, including idecabtagene vicleucel for MM, lisocabtagene maraleucel for large B-cell lymphoma, tisagenlecleucel for ALL and large B-cell lymphoma, brexucabtagene autoleucel for MCL, and axicabtagene ciloleucel for FL and large B-cell lymphoma. Despite its potential, CAR-cell therapy faces significant challenges, including neurotoxicity, cytokine release syndrome, and off-tumor toxicity. These limitations not only hinder its efficacy but also introduce unwanted side effects. Therefore, urgent research is needed to unravel the underlying molecular mechanisms and overcome these obstacles.

**TABLE 2:** CAR cell therapy against HMs in clinical trial (from www.clinicaltrials.gov).

NCT number	Target	CAR cell	Status	Phase	Conditions
NCT05563545	CD19	CAR-NK	Completed	Phase 1	ALL
NCT03692767	CD22	CAR-NK	Unknown	Early Phase 1	Refractory B-Cell Lymphoma
NCT06045091	BCMA	CAR-NK	Recruiting	Early Phase 1	MM, Plasma Cell Leukemia
NCT05574608	CD123	CAR-NK	Recruiting	Early Phase 1	AML Refractory; AML Recurrent
NCT05008575	CD33	CAR-NK	Unknown	Phase 1	R/R AML
NCT05734898	NKG2D	CAR-NK	Recruiting	Not Applicable	AML
NCT03824964	CD19/CD22	CAR-NK	Unknown	Early Phase 1	Refractory B-Cell Lymphoma
NCT05667155	CD19/CD70	CAR-NK	Recruiting	Phase 1	B-NHL
NCT05215015	CD33/CLL1	CAR-NK	Unknown	Early Phase 1	AML
NCT05487651	CD19	CAR-T	Recruiting	Phase 1	B-cell Lymphoma
NCT05941156	CD56	CAR-T	Recruiting	Phase 2	Extranodal NK/TCL; NK-Cell Leukemia
NCT03049449	CD30	CAR-T	Completed	Phase 2	Lymphoma
NCT04033302	CD7	CAR-T	Unknown	Phase 1/2	T-ALL, TCL, NKL, AML
NCT05487495	CD5	CAR-T	Recruiting	Phase 1	T-Cell ALL; Lymphoma
NCT05948033	CD70	CAR-T	Recruiting	Phase 1/2	Lymphoma
NCT05773040	CD79b	CAR-T	Recruiting	Phase 1	Lymphomas; B-cell Lymphomas
NCT04049383	CD19/CD20	CAR-T	Recruiting	Phase 1	R/R ALL

### 2.6 Combined treatment

Clinical studies demonstrated that combining different clinical strategies is more effective by avoiding the pitfalls of a single treatment. The combination of mutated IDH inhibitors with induction and consolidation chemotherapy improved the overall survival rates of AML patients (Stein et al., 2021). The combination of vorinostat with chemotherapy and rituximab was available in HIV-related B-Cell NHL with high-risk features in clinical trials (Ramos et al., 2020). The combination of chemotherapy, HSCT, and tyrosine kinase inhibitors significantly improved the event-free survival rate from 20% to 80% in Philadelphia (Ph) chromosome-positive ALL (Shimada, 2019). A phase II clinical trial investigated the combination of anti-CD30 CAR-T treatment with a PD-1 inhibitor in R/R CD30-positive lymphoma. Of the 12 evaluated patients, the trial reported an impressive overall response rate of 91.7% and a complete response rate of 50%. Furthermore, 63.6% patients who got a response after CAR-T therapy sustained their response throughout the follow-up period (Sang et al., 2022). Anas Younes et al. made the antitubulin agent monomethyl auristatin E (MMAE) attach to a CD30-specific monoclonal antibody by an enzyme-cleavable linker, producing the antibody-drug conjugate (ADC) brentuximab vedotin (SGN-35) to enhance the antitumor activity of CD30-directed therapy (Younes et al., 2010). Other ADCs like gemtuzumab ozogamicin, a selective anti-CD33 antibody-calicheamicin, was approved for clinical practice (Appelbaum and Bernstein, 2017), and inotuzumab ozogamicin (INO) as ADC that consists of a humanized anti-CD22 monoclonal antibody linked to calicheamicin, was also approved for clinical practice for B-ALL patients (Kantarjian et al., 2016). Compared with single targeted therapy, the combination of BRD4 inhibitor (JQ1) and HMA (azacitidine) was more effective for inducing MDS and AML cell apoptosis (Pericole et al., 2019). FDA approved Polatuzumab Vedotin-piiq (Polivy, Palotuzumab) in combination with rituximab + cyclophosphamide, doxorubicin, and prednisone, first-line treatment of patients with diffuse large B-cell lymphoma in 2023.

## 3 The application of nanocarriers-mediated drug delivery

**FIGURE 4:**
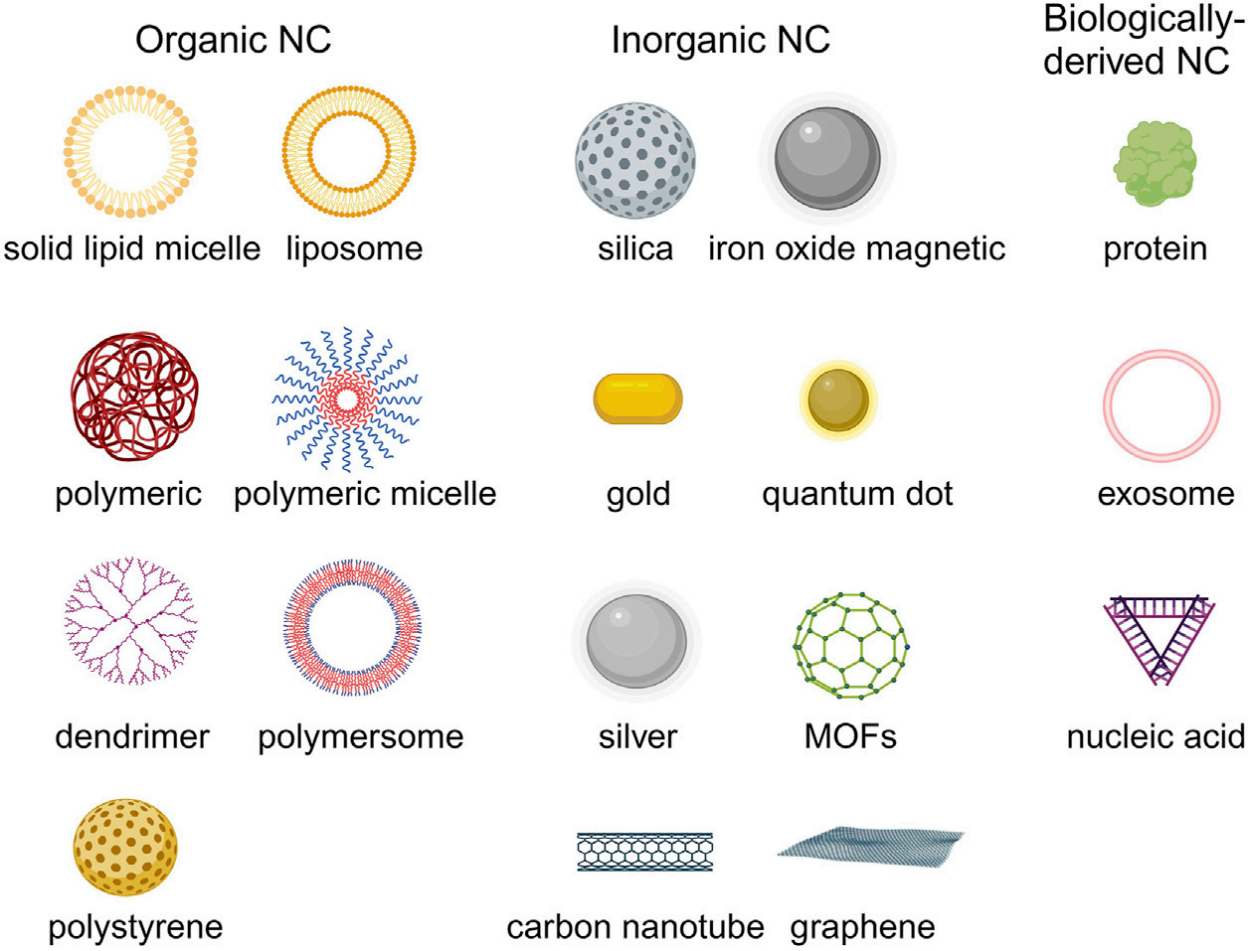
The common nanocarriers for drug delivery in HMs treatment. Created by BioRender (agreement number: TS26D15BJS).

Due to the physical and chemical properties of the drugs, their clinical effectiveness is limited. Nanodrug delivery systems can overcome drug delivery problems in the body. The common types of nanocarriers for drug delivery in HMs therapy include organic, inorganic, and biological-derived nanocarriers (Figure 4) (Hani et al., 2023). Currently, the FDA-approved or clinically researched nanomedicine for treating HMs primarily employs organic nanomaterials, such as liposomes and polymer micelles. Inorganic nanomaterials, biological-derived nanomaterials, and most of organic nanomaterials for drug delivery in treatment of HMs are still in preclinical studies (Li J. et al., 2023).

### 3.1 Organic nanocarriers

Organic nanocarriers mainly include liposome, solid lipid micelle, polymeric nanoparticle, polymeric micelle, polymersome, polystyrene and dendrimer, etc (Kowalczuk et al., 2014; Peng et al., 2020).

#### 3.1.1 Lipid based nanocarriers

Lipid-based nanocarriers include solid lipid micelle (SLM) and liposome. SLM is composed of a single layer of lipid molecules, and the main application of SLM is in the delivery of hydrophobic drugs by solubilizing these drugs within their lipid core (da Rocha et al., 2020). The utility of doxorubicin (DOX) and vincristine (VCR) co-encapsulated SLM to treat B-cell lymphomas had obtained good anti-cancer efficacy in lymph cancer animal model (Dong et al., 2016). Liposome is the earliest and most successful application for drug delivery in HMs treatment. Liposome compositions are phospholipids and cholesterol, which form a metastable spherical structure in an aqueous solution with a lipid bilayer that includes a membrane surrounding the internal aqueous compartment. This structure is stabilized by the cooperation of hydrophobic interactions, hydrogen bonding, van der Waals forces, and electrostatic interactions. Because liposome has hydrophobic properties in the lipid bilayer and hydrophilic properties in the aqueous compartment, it can simultaneously encapsulate hydrophobic and hydrophilic drugs. In addition, the addition of cholesterol in liposome helps stabilize the membrane bilayer and prevents leakage of drugs encapsulated in the aqueous core. There are some FDA-approved liposomal drugs for HMs treatment in the clinic, including liposomal doxorubicin (Doxil^®^/Caelyx^®^/LipoDox^®^) treating MM and lymphoma, liposomal daunorubicin (DaunoXome^®^) treating AML and non-Hodgkin lymphoma (NHL), liposomal vincristine (Marqibo) treating ALL and NHL, and liposomal cytarabine + daunorubicin (VYXEOS^®^) treating AML. Parts of liposomal drugs in the clinical trial stage are shown in Table 3. Compared to traditional free drugs, those liposomal drugs exhibit superior pharmacokinetic attributes, delivering more substantial clinical advantages to patients and significantly enhancing the clinical therapeutic effect (Feldman et al., 2012). In addition, the liposome can also be utilized to mimic immune cell membranes like natural killer cells by embedding functional proteins or ligands to kill HM cells (Zeinabad et al., 2023).

**TABLE 3:** Liposomal drugs against HMs in clinical trial (from www.clinicaltrials.gov).

NCT number	Nanocarrier	Drug	Developed stage	Conditions
NCT04802161	Liposome	Daunorubicin + Cytarabine	Phase 2	AML, AML Post Cytotoxic Therapy; AML with multilineage dysplasia, CML, MDS
NCT00389428	Liposome	Daunorubicin + Cytarabine	Phase 1 (completed)	Hematologic Neoplasms
NCT00005942	Liposome	Daunorubicin citrate	Phase 1/Phase 2 (completed)	CML, previously treated MDS, recurrent adult AML
NCT00418951	Liposome	Amphotericin B	Phase 2 (completed)	AML, MDS
NCT05857982	Liposome	Mitoxantrone hydrochloride	Phase 1/Phase 2	R/R MM

#### 3.1.2 Polymeric nanocarrier

Polymeric nanocarriers are produced from a polymeric material and are solid and colloidal particles with sizes ranging from 1 to 1,000 nm, possessing diverse structures and morphologies (Xiao et al., 2022). According to the structure difference, the types of polymeric nanocarriers are classified as polymeric nanoparticle-like, polymeric micelle, and polymersome (Kuperkar et al., 2022). Polymeric nanoparticles like poly(lactic-co-glycolic acid) are prepared by hydrophobic polymer materials in water solutions by phacoemulsification or other methods (Astete and Sabliov, 2006; Danhier et al., 2012). The behavior of amphiphilic block or graft copolymers is similar to that of conventional amphiphilic substances, and these polymers form polymeric micelles in aqueous solutions above the critical micelle concentration (Mourya et al., 2011). Polymersomes are prepared from amphiphilic polymers and are more stable than liposomes (Anajafi and Mallik, 2015). Polymersomes enable the loading of water-soluble drugs in hydrophilic inner lumen and loading lipophilic cargo in the hydrophobic part of the polymersomes’ membrane (Bleul et al., 2015). The advantages of polymeric nanocarriers include the high stability to protect drug molecules with biological activity against the environment, the high drug loading efficiency and the ability to control drug release in cancer lesions (Cano et al., 2019; Cano et al., 2020).

Pegaspargase (oncaspar) is a covalent conjugate of polyethylene glycol and asparaginase for the first-line treatment of ALL (Heo et al., 2019). Many other pegylated drugs and pegylated liposomal drugs are in clinical trials for treating HMs (NCT02526823, NCT02001818, NCT00573378). Eunji Kwak et al. used poly-L-lysine (PLL) based polymeric nanoparticle to load siBCL2 to treat non-Hodgkin’s lymphoma (NHL) (Kwak et al., 2022). Neha Mehrotra PhD et al. constructed a polylactic acid (PLA)-based block copolymeric nanocarrier for the co-delivery of navitoclax and decitabine for AML therapy (Mehrotra et al., 2023). Wen Bao et al. used daunorubicin-loaded poly(lactic-co-glycolic acid)-poly-l-lysine-polyethylene glycol (PLGA-PLL-PEG)-transferrin nanoparticles achieved effective anti-leukemia treatment *in vitro* and *in vivo* (Bao et al., 2019). Donato Cosco et al. encapsulated miR-34a into chitosan/PLGA nanoparticles against multiple myeloma *in vitro* and *in vivo* (Cosco et al., 2015). Yinan Zhong et al. applied triple-block polymer poly(ethylene glycol)-b-poly(trimethylene carbonate-co-dithiolane trimethylene carbonate)-b-spermine (PEG-P(TMC-DTC)-SP) forming polymersome to encapsulate granzyme B and achieved human multiple myeloma killing in mice model (Zhong et al., 2020).

### 3.2 Inorganic nanocarriers

The flexible preparation options, excellent biocompatibility, adjustable size, and unique physicochemical properties make the inorganic nanomaterials popular application in imaging and drug delivery in cancer therapy (Wang et al., 2016). Furthermore, some inorganic nanoparticles can self-made medicine to kill cancer cells (Adamo et al., 2023; Li Z. et al., 2023). Inorganic nanocarriers are prepared by inorganic materials such as silica and metal materials, etc. The types of inorganic nanomaterials used for drug delivery in HMs treatment, mainly include silicon dioxide nanoparticle (NP), gold nanoparticle, silver (Ag) NP, calcium NP, carbon nanotube, quantum dot, metal-organic framework (MOF) and graphene and metal oxide (Fe_3_O_4_, ZnO, CuO), etc (Ghosn et al., 2019; Huang H. et al., 2020). Their detailed applications in preclinical HMs treatment are shown in Table 4.

**TABLE 4:** Application of inorganic nanocarriers in HMs treatment.

Type of inorganic nanocarrier	Drug	Therapeutic strategy	Type of HMs	Model	References
Carbon nanotube	Doxorubicin (DOX)	Chemotherapy	Chronic myelogenous leukemia	*In vitro*	Li et al. (2010)
	DOX	Chemotherapy	Burkitt’s Lymphoma	*In vitro* *+* *In vivo*	Falank et al. (2019)
Calcium phosphosilicate NP	Indocyanine green	Photodynamic therapy	Leukemia stem cells	*In vitro* *+* *In vivo*	Barth et al. (2011)
Mesoporous silica NP	Gemcitabine	Chemotherapy	REH leukemia cells	*In vitro*	Durfee et al. (2016)
	Anthracycline daunorubicin	Chemotherapy	Leukemia stem cells	*In vitro* *+* *In vivo*	Mandal et al. (2018)
Fe_3_O_4_@SiO_2_	Cytarabine	Chemotherapy	Promyelocytic leukemia cells	*In vitro*	Shahabadi et al. (2016)
Gold NP	FMS-like tyrosine kinase 3 inhibitor	Targeted therapy	Acute myeloid leukemia	*In vitro*	Simon et al. (2015)
ZnO NP	ZnO nanoparticle	Autophagy	Multiple myeloma	*In vitro*	Li et al. (2023b)
Cadmium telluride quantum dot	DOX	Chemotherapy	Extramedullary multiple myeloma	*In vitro*	Chen et al. (2018)
Ag NP	Ag nanoparticle + Anti-CD20 antibody	Targeted therapy	Chronic lymphocytic leukemia	*In vitro*	Adamo et al. (2023)
Graphene oxide	Rituximab	Targeted therapy	Non-Hodgkin lymphoma	*In vitro* *+* *In vivo*	Luo et al. (2016)
Bimetallic MOF	Azacitidine + pro-autophagic Beclin-1 peptide	Chemotherapy + autophagy	leukemic blasts; leukemia stem cell	*In vitro* *+* *In vivo*	Song et al. (2023)

### 3.3 Biologically-derived nanocarriers

Biologically-derived nanocarriers are an emerging type of drug carrier in biomedicine due to their low cost, unique physicochemical properties, biological functions, and excellent biocompatibility (Sun et al., 2021). Biologically-derived nanocarriers can be extracted from various organisms such as plants, algae, bacteria, fungi, actinomycetes, and yeast, etc (Singh et al., 2016; Karunakaran et al., 2023). The current common types of biologically-derived nanocarriers mainly include proteins, exosomes, and nucleic acids (Boyiadzis and Whiteside, 2017; Curley and Cady, 2018; Ma et al., 2021).

Proteins as the drug delivery carrier have a long history in cancer therapy (Hawkins et al., 2008; Jain et al., 2018). Paras Famta et al. applied human serum albumin (HSA) to encapsulate ibrutinib (IB-NPs), and this IB-loaded albumin nanoparticle completed significant leukemic K562 cellular killing (Famta et al., 2023). Ehsan Vafa et al. utilized the bovine serum albumin protected gold nanozymes (BSA-Au nanozymes) as a nanodrug for the treatment of acute T-type lymphoblastic leukemia (Jurkat) by production of excessive ROS and effect on the expression of anti-apoptotic genes (Bcl-2) (Vafa and Bazargan-Lari, 2021). To satisfy the demand for drug delivery, DNA, a nucleic acid material, has been designed with different shapes and sizes based on the classic Watson-Crick base-pairing for molecular self-assembly (Ma et al., 2021). Patrick D. Halley et al. successfully utilized DNA nanostructures to circumvent daunorubicin drug resistance at clinically relevant doses in a leukemia cell line model (Halley et al., 2016). The exosome is the most popular biologically-derived nanocarrier in cancer therapy in recent years (Chinnappan et al., 2020; Allegra et al., 2022). Exosomes are small (30–150 nm) membranous vesicles of endocytic origin containing cytokines, RNAs, growth factors, proteins, lipids, and metabolites, and can be produced by all cells under physiological and pathological conditions (Boyiadzis and Whiteside, 2017; Allegra et al., 2022). Qing-Hua Min et al. successfully applied exosomes mediating a horizontal transfer of drug-resistant trait in chronic myeloid leukemia cells by delivering miR-365 (Min et al., 2018). Qinhua Liu et al. used iRGD-targeted exosomes produced by engineered immature dendritic cells expressing exosomal membrane protein (Lamp2b) to load BCL6 siRNA by electroporation (Liu Q. et al., 2022). The result showed the iRGD-Exo loaded with BCL6 siRNA suppressed diffuse large B-cell lymphoma cell proliferation *in vitro* and inhibited tumor growth *in vivo*.

## 4 The types and applications of cell membrane camouflage on nanodrugs

**FIGURE 5:**
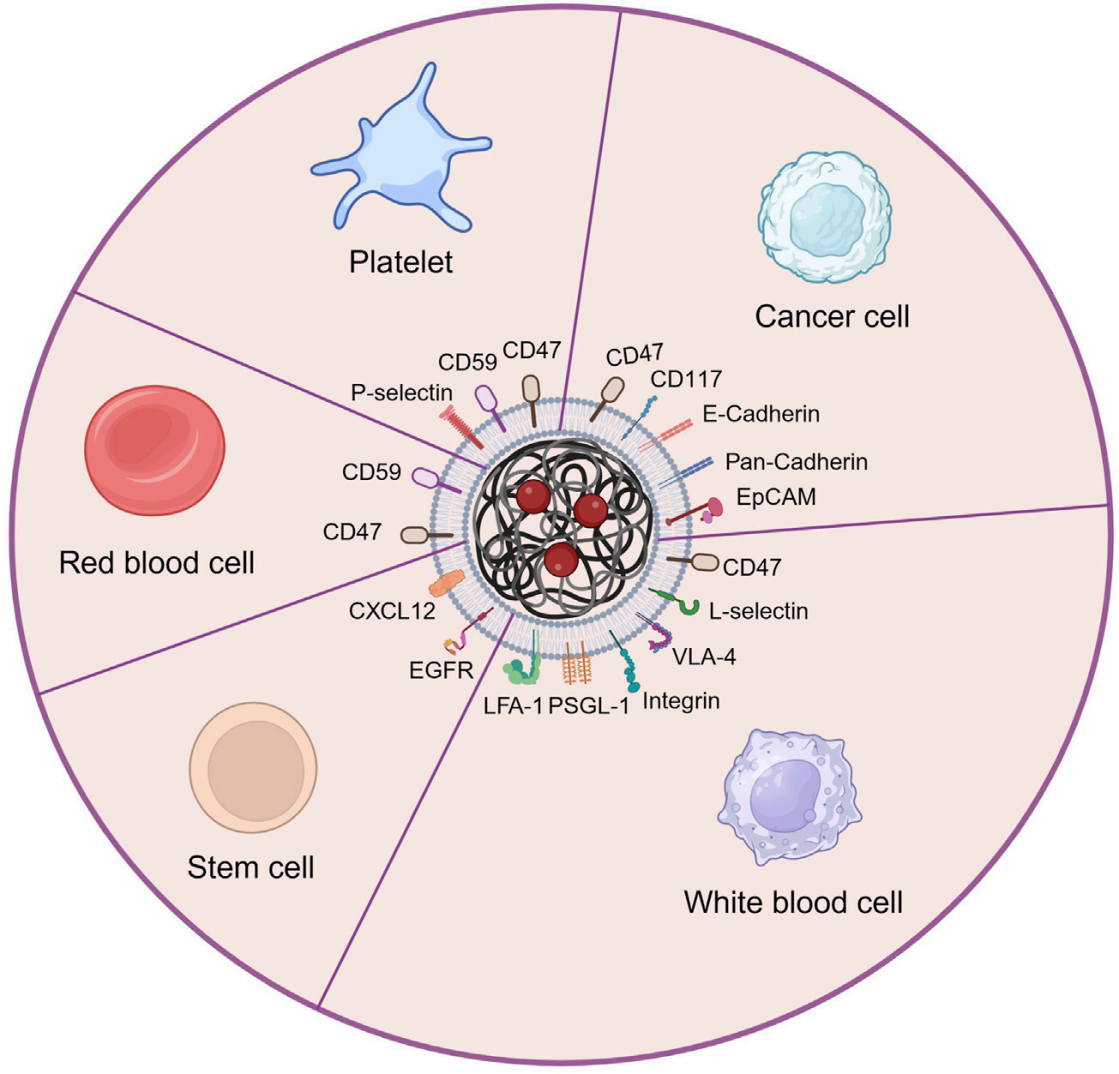
The main types of cell membrane and membrane proteins used for camouflaging nanodrug in HMs treatment. Created by BioRender (agreement number: NT26D16T7T).

Lacking of active targeting ability weaken the delivery efficacy of the nanodrug delivery system (Wang and Thanou, 2010). Nowadays, targeting decoration using specific ligands or natural cell membrane fragments on the surface of nanodrugs can significantly increase the accumulation of nanodrugs in HM lesions, enhancing therapeutic efficiency (Ahmad et al., 2021). Better than targeting ligands using antibodies, peptides, aptamers, or small molecular, biomimetic decoration endows nanodrug with more functions, including preventing of drug leakage, target specificity and unique homing abilities to HM lesions, low toxicity and immune evasion, etc (Aryal et al., 2013; Zhang et al., 2020; Zhao et al., 2022). Common biomimetic methods include cell membrane camouflage on the surface of nanodrugs, cellular Trojan horses loading nanodrugs in intact cells and attaching nanodrugs to cell surfaces (Powsner et al., 2021). Different source cells can be used for camouflage nanodrugs, including red blood cells (RBCs), platelets, bacteria, cancer cells (CCs), stem cells (SCs), and white blood cells (WBCs), which are coated onto nanodrugs by co-extrusion, extrusion/sonication, freeze-thaw/sonication, extrusion/sonication and stirring, and more (Choi et al., 2020). In this review, we focus on a cell membrane-wrapped nanodrug delivery system, and will detailly introduce the types of various cell membrane and their mechanism and difference in application (Labernadie et al., 2017; van Manen et al., 2019; Wang et al., 2019; Zhao et al., 2021b; Harris et al., 2022; Kong et al., 2022; Wu et al., 2022; Chen et al., 2023) (Figure 5; Table 5).

**TABLE 5:** Preclinical application of cell membrane camouflaged nanodrugs in HMs treatment.

Source of cell membrane	NC	Targeting interaction	Drug	Therapeutic strategy	Type of HMs	Model	References
RBC	PLGA	no	DOX	Chemotherapy	Lymphoma	*in vitro*	Luk et al. (2016)
Platelet	W_18_O_49_	P-selectin/CD44	Metformin	Photodynamic/Photothermal therapy	Burkitt’s lymphoma	*in vitro* + *in vivo*	Zuo et al. (2018)
CC	MSNs	Tumor-associated antigen (Pan-cad and E-cad) + Homotypic targeting	Isoimperatorin	Traditional Chinese medicine therapy	NHL	*in vitro* + *in vivo*	Zhao et al. (2021b)
	PCL-PEG-PCL	Homologous targeting	Bortezomib	Targeting therapy	MM	*in vitro* + *in vivo*	Qu et al. (2022)
WBC	MSNs	CD47/signal regulatory protein α (SIRPα)	DOX + Shanzhiside methylester	Chemotherapy + Traditional Chinese medicine therapy	NHL	*in vitro* + *in vivo*	Zhao et al. (2021a)
SC	PLGA	CXCL12/CXCR4	DOX + perfluorobromooctane + Pt	Chemotherapy	AML	*in vitro* + *in vivo*	Kong et al. (2022)

### 4.1 Red blood cell membrane (RBCM)

Red blood cell (erythrocyte) membrane has been widely used for wrapping nanodrug attributed to its good biocompatibility, biodegradability, and long circulating time in the body (Rao et al., 2015; Xia et al., 2019; Nguyen et al., 2023). Liangfang Zhang’s team first developed and applied RBCM to coat nanodrug for HMs therapy. The RBCM-cloaked NPs loaded with DOX enabled reach tumor lesions through the enhanced permeability and retention (EPR) effect. Consequently, this nanodrug demonstrated superior toxicity compared to free DOX when treating AML cells (Aryal et al., 2013) and EL4 mouse lymphoma cells *in vitro* (Luk et al., 2016). However, lacking specific targeting ability limited the direct application of RBCM in nanodrug delivery systems (Zhang et al., 2021).

### 4.2 Platelet membrane (PM)

Platelet is another popular type of blood cell and chosen for extracting cell membranes to camouflage nanodugs (Zhang et al., 2018). Similar to RBCM (Zou et al., 2019), PM has an inherent immune escaping ability attributed to the CD47 and CD59 protein in PM sending “do not eat me” signals to immune cells (van Manen et al., 2019; Wang et al., 2019). Advanced to RBCM, PM has specifically cancer-targeting ability attributed to the overexpression of P-selectin, which allows PM-cloaked nanodrug to selectively bind to the CD44 receptors upregulated on the surface of cancer cells (Wang et al., 2019). Huaqin Zuo et al. built platelet-mimicking nanoparticles co-loaded with W_18_O_49_ and metformin (Met) to alleviate tumor hypoxia (PM-W_18_O_49_-Met NPs) (Zuo et al., 2018). The result exhibited that platelet membranes not only protected W_18_O_49_ from oxidation and immune evasion but also increased the accumulation of W_18_O_49_ in tumor sites via the active adhesion between platelets and lymphoma cells compared with non-platelet membrane-coated W_18_O_49_-Met NPs. Under the cooperation of W_18_O_49_ and metformin, the reactive oxygen species and heat generation significantly increased in lymphoma cells, hence inducing cellular death *in vitro* and tumor growth inhibition *in vivo* by enhanced photodynamic and photothermal therapy.

### 4.3 Cancer cell membrane (CCM)

The cancer cell membrane camouflage has considerable advantages in nanodrugs-mediated cancer targeting therapy because of their inherent abilities of targeting and homing to cancer lesions (Labernadie et al., 2017), or even activating immune therapeutic function to cancer (Fang et al., 2014; Guo et al., 2022; Johnson et al., 2022), expecting of prolonging blood circulation time of nanodrugs (Fang et al., 2014; Yaman et al., 2020). Qiangqiang Zhao et al. constructed OCI-LY10 cancer cell membrane (CCM) coated mesoporous silica nanoparticles (MSNs) loaded with the traditional Chinese medicine isoimperatorin (ISOIM), which was called CCM@MSNs-ISOIM (Zhao et al., 2021b). The CCM@MSNs-ISOIM not only exhibited high targeting and adhesion to OCI-LY10 cells by Ca^2+^-dependent proteins including Pan-Cadherin and E-Cadherin, but also significantly induced OCI-LY10 cytotoxicity in cellular level experiment and subcutaneous OCI-LY10 cell lymphoma model in nude mice. In addition, the safety study in mice blood and organs illustrated the excellent biocompatibility of CCM@MSNs-ISOIM. Jenna C. Harris et al. encapsulated DOX into PLGA nanocarriers which were coated with cytoplasmic membranes derived from human AML cells (Harris et al., 2022). Compared to DOX-loaded PEG-coated nanodrugs, DOX-loaded AML cell-coated nanodrugs exhibited significantly higher AML cell killing at the same drug concentration, which demonstrates the effective targeted accumulation effect mediated by homology targeting of CD117 from AML cell membrane. Daniel T. Johnson et al. fabricated AML cell membrane-coated and immunostimulatory adjuvant (CpG)-loaded nanoparticle (AMCNP) as the cancer vaccine to treat AML (Johnson et al., 2022). Under the double stimulation of cancer-associated antigens and immunostimulatory adjuvants, the antigen-presenting cells (APCs) were activated, finally stimulating functional leukemia-specific T cell responses to target and kill AML cells.

### 4.4 White blood cell membrane (WBCM)

White blood cells (leukocytes) membrane camouflaged nanodrugs are also very available in cancer therapy because they are capable of passing through blood vessels, targeting tumor sites, and avoiding immune attacks (Oroojalian et al., 2021). Better than other kinds of cell membranes, WBCM can assist nanodrugs actively across endothelial cells by the interaction of selectins (on endothelial cells) and their ligands (on WBCM) such as P-selectin glycoprotein ligand-1 and L-selectin (Si et al., 2016; Poudel et al., 2020). In addition, the expressed P-selectin glycoprotein ligand-1 (PSGL-1), L-selectin, lymphocyte function-associated antigen 1 (LFA-1), integrin, or very late antigen-4 (VLA-4) on the WBCMs increase their cell adhesion with the surface receptors of cancer cells and hence achieving active targeting function (Wu et al., 2022; Chen et al., 2023). The common WBCMs to wrap nanodrugs are usually from macrophages, neutrophils, natural killer (NK) cells, or T cells, etc (Wang et al., 2022). Neutrophils are recognized as the first responders to tissue injury and signs of chronic inflammation like tumor microenvironment. DOX and hanzhiside methylester (SM) co-loaded mesoporous silica nanoparticles (MSNs) coated by a neutrophil membrane (Nm@MSNs@(DOX/SM)) was constructed by Xianjun Ma’s research team (Zhao et al., 2021a). The *in vivo* anti-tumor study showed that this biomimetic nanodrug evaded immune recognition, actively accumulated in the non-Hodgkin’s lymphoma site, and released drugs in the tumor site to inhibit the inflammation of tumor microenvironment by SM and further enhanced the anti-tumor effect by DOX.

### 4.5 Stem cell membrane (SCM)

Stem cell membranes also have camouflaged value for nanodrugs in HMs therapy because of containing a large number of molecular recognition moieties, which play a crucial role in interactions with tumor cells, hence exhibiting a natural and high tumor affinity (Gao et al., 2016a; Gao et al., 2016b). Some bone marrow-derived steam cells such as bone marrow stromal cells (Kong et al., 2022) and leukemia stem cells (Song et al., 2023), have an inherent ability to recognize and combine with HMs, therefore their cell membrane can also be utilized for wrapping nanodrugs to target to and kill HM cells. Fei Kong et al. used bone marrow stromal cell membrane coat oxygen-carrying nanoparticle conjugated with ultra-small nanozyme (PFOB@PLGA@Pt@DOX-CM) (Kong et al., 2022). The PFOB@PLGA@Pt@DOX-CM could be actively internalized by the leukemia cells in the blood and released the loaded DOX and Pt nanozyme inside the leukemia cells to achieve synergistic antitumor efficacy. Meanwhile, the adhesive properties of the stromal cell membrane enabled the PFOB@PLGA@Pt@DOX-CM to home to the bone marrow and to kill the retained leukemia cells. Therefore, this nano-formulation possessed a synergistic anti-AML efficacy via integrating chemotherapy, nanozyme-induced cascade catalytic activity, and CXCR4 antagonism. More importantly, the bone marrow stromal cell membrane also acted as a CXCR4 antagonism to block the CXCR4/CXCL12-mediated homing of leukemia cells to the bone marrow and infiltration to other organs. Yue Song et al. applied leukemia stem cell linked with pro-autophagic Beclin-1 peptide to wrap bimetallic metal-organic framework (Mn^2+^/Fe^3+^ MOF) loading DNA hypomethylating agent (azacitidine, AZA), which is named AFMMB (Song et al., 2023). The AFMMB selectively targeted the leukemic blasts due to the homing ability of the leukemia stem cell membrane. Following internalization, AFMMB accumulated in the Golgi apparatus by binding to the Golgi-associated plant pathogenesis-related protein 1 and triggered autophagy, leading to its disintegration and release of Fe^3+^, Mn^2+^, and AZA. While AZA and Mn^2+^ restored the stimulator of interferon genes pathway by inhibiting DNA methylation, accumulation of Fe^3+^ inactivated the endogenous iron-dependent m^6^A demethylase, thereby increasing global m^6^A RNA modification and suppressing PD-L1. The dual epigenetic action enhanced the antigenicity of the AML cells by upregulating MHC-I molecules and downregulating PD-L1, resulting in enhanced T-cell-mediated immune response and further killing AML cells.

### 4.6 The combined application of cell membrane camouflage with ligands

To endow some cell membranes like RBCM targeting ability, or further enhance the targeting function of cell membrane camouflaged nanodrug to endothelial cells or cancer lesions, some ligands are usually embedded into cell membrane. Fangrong Zhang et al. applied CD38-targeting peptide (P) embedded RBCM wrap spherical porous hollow Trinickel monophosphide nanoparticles (Ni3P NPs) loaded with bortezomib (BTZ) to form P-R@Ni3P-BTZ nanodrug (Zhang et al., 2023). *In vitro* and *in vivo*, it proved that P-R@Ni3P-BTZ has excellent targeting ability to CD38^+^ MM cells and is highly effective in killing MM cells by cooperating with photothermal therapy by Ni3P and chemotherapy by BTZ. Zhen Gu’s research team constructed alendronate (Ald) targeting modification and platelet membrane-coated nanoparticulate platform for targeted delivery of BTZ at the myeloma site based on the bone microenvironment and myeloma cell sequential targeting strategy (Hu et al., 2016). In addition, tPA was decorated on the platelet membrane via biotin-streptavidin affinity to dissolve the thrombus readily and reduce the mortality of multiple myeloma patients. Ald helped tPA-Ald-PM-NP@BTZ accumulate at the bone site by chelating calcium ions rich in the bone microenvironment, decreasing the off-target effects. Furthermore, platelet membrane helped tPA-Ald-PM-NP@BTZ achieve secondary targeting from bone microenvironment to myeloma cells by specific affinity between P-Selectin on PM and CD44 receptor on myeloma cells. Finally, compared with other BTZ formulations, the tPA-Ald-PM-NP@BTZ achieved excellent anti-multiple myeloma efficacy in the multiple myeloma-bearing Nod-SCID mice and significantly prolonged the survival time of mice.

## 5 Conclusion and outlook

The physical and chemical properties of drugs limited their clinical application in treating HMs. Nanocarriers from different sources such as organic materials, inorganic materials, biological-derived materials, and their combination, have been largely and successfully used to resolve drug delivery problems in HMs therapy. Some liposome-based nanodrugs like Doxil^®^ have been allowed for marketing by the FDA to patients with HMs. Even though the ERP effect allows nanodrugs to pass through broken blood vessels, lacking targeting decoration limits their active accumulation and deep penetration in tumor tissues (Subhan et al., 2021). In addition, non-targeting decorated nanodrugs are hard to recognize and combine with non-solid tumors like leukemia (Huang et al., 2019). Common targeting decoration includes modifying ligands like antibodies or cell membrane fragments on the surface of nanodrugs, or delivering nanodrugs using intact cells like immune cells by loading nanodrugs into cells or linking nanodrugs on cellular surface (Alghamdi et al., 2022).

Compared with targeting ligands, which only administrate targeting function, cell membrane camouflage not only assists nanodrugs in actively targeting to cancer sites but also enhances the biocompatibility and prolongs the blood circulation time of nanodrugs in the body, hence further improving therapeutic efficiency (Zhu et al., 2022). For HMs therapy, the main sources of cell membrane fragments are red blood cells, platelets, cancer cells, white blood cells, and stem cells. When extracting cell membrane fragments and preparing cell membrane-wrapped nanodrugs, membrane proteins are normally reserved by intelligent technologies. This is also the main reason for keeping targeting and biocompatible functions similar to intact cells (Chugh et al., 2021). Because red blood cells have no targeting ability to tumors, their membrane is usually combined with targeting ligands to co-modify nanodrugs. CCM, WBCM, and SCM have unique targeting abilities and adhesion to cancer cells because of homology targeting, immune recognition and self-updating requirements, respectively. Furthermore, some of CCM, WBCM, and SCM have the ability to activate immune responses to HMs, hence producing combined therapy with drugs. This “kill two birds with one stone” strategy that targets accumulation and immune activation mediated by cell membrane demonstrates huge advantages of cell membrane camouflage in treating HMs and other cancers.

Nowadays, even though a number of studies about (cell membrane camouflaged) nanodrug delivery system have been experimental detection in the treatment of HMs in mice models and enable significantly improve therapeutic efficiency compared with free drugs, few studies of nanodrugs are being conducted in the clinical process. Quantitative production problems, expensive expenses, and unknown long-lasting and cumulative toxicity of nanocarriers and cell membranes in the body are the main restrictions for clinical application (Xuan et al., 2023). Further laboratory and clinical trials are imperative to ensure the safety profile of nanodrugs. Additionally, hybrid cell membrane camouflage and a combination of cell membranes with ligands have been utilized for improving cancer therapeutic efficiency attributed to their versatility (Rao et al., 2020). In the future, these strategies should be explored and applied for HMs therapy so that avoiding pitfalls caused by single decoration.

In summary, with the help of various nanocarriers and biomimetic camouflage using cell membrane fragments, drugs can be successfully and highly effectively delivered into HM lesions. These studies supply more chances for the clinical application of drugs in HMs and other cancers.
